# The surgical learning curve for percutaneous Zadek osteotomy for treatment of insertional achilles tendinopathy

**DOI:** 10.1007/s00402-024-05405-3

**Published:** 2024-06-26

**Authors:** SarahRose Hall, Jonathan R. M. Kaplan, Tammy Phillips, J. Benjamin Jackson, Ettore Vulcano, Tyler A. Gonzalez

**Affiliations:** 1 University of South Carolina, School of Medicine, 6311 Garners Ferry Rd, Columbia, SC 29209 USA; 2 Duke University Orthopedics, 200 Trent Dr, Durham, NC 27710 USA; 3 University of Florida Orthopedics, 3450 Hull Rd, Gainesville, FL 32607 USA; 4 Prisma Health Orthopedics – Lexington, 104 Saluda Pointe Drive, Lexington, SC 29072 USA; 5 Columbia University Division of Orthopedics at Mount Sinai Medical Center, 4302 Alton Rd, Simon Building, Suite 220, Miami Beach, FL 33140 USA

**Keywords:** Haglund syndrome, Insertional achilles tendinopathy, Learning curve, Minimally invasive surgery, Zadek osteotomy

## Abstract

**Introduction:**

The Zadek Osteotomy has been described as an effective technique for the treatment of insertional Achilles tendinopathy. Recently, this strategy has been modified using minimally invasive techniques. A learning curve has been observed in many minimally invasive procedures in foot and ankle surgery. This retrospective study first intended to evaluate if there is a learning curve associated with the percutaneous Zadek Osteotomy. Further, if a learning curve was observed, we planned to assess the data for associated changes in complications and postoperative outcomes.

**Methods:**

A retrospective analysis of 98 patients who underwent percutaneous Zadek Osteotomy was performed. Patient charts were reviewed for operative times, complications, union rates, and Foot Function Index (FFI) and Visual Analogue Scale (VAS) scores. Analysis of variance was utilized to assess for differences between groups of cases.

**Results:**

Patients included 61 females and 37 males. Mean age was 51.28 ± 11.12 (range 28–81) years. Mean follow-up time was 42.07 ± 12.99 (range 24–65) months. Significant increases in operative times were observed in cases 1–14 when compared to cases 15–98 (*p* < 0.001). Improvements in FFI and VAS scores were observed at final follow-up within each case group (*p* < 0.001); there were no differences detected in FFI or VAS scores between groups of cases. There was no difference detected in number of complications between intervals of cases.

**Conclusion:**

A learning curve was observed for the percutaneous Zadek Osteotomy, which was overcome around case 14. This learning curve was only observed in terms of procedure length. A surgeon’s level of inexperience with the technique does not appear to affect functional outcomes, nonunion, or need for revision.

**Level of evidence IV:**

Data will not be deposited in a repository.

**Supplementary Information:**

The online version contains supplementary material available at 10.1007/s00402-024-05405-3.

## Introduction

Insertional Achilles tendinopathy (IAT) is one of the most common pathologies treated by orthopaedic foot and ankle surgeons. First line of treatment for this pathology includes conservative management strategies such as anti-inflammatory medication, physical therapy, pulsatile ultrasound therapy, extracorporeal shockwave therapy, and footwear modification. However, these nonoperative approaches fail in 20–40% of cases [1]. The traditional surgical approach for intractable IAT consists of open tendon debridement, posterosuperior calcaneus exostectomy, and reinsertion of the Achilles tendon. The Zadek Osteotomy (ZO), a dorsal closing wedge calcaneal osteotomy, has also been described as a successful procedure for the treatment of IAT [1, 2]. 

More recently, this strategy has resurfaced in the literature as a minimally invasive (MIS) techniques [2–6]. Generally, MIS has been associated with lower postoperative complications, better cosmesis, and less postoperative pain [2, 7–10]. However, with new MIS procedures a learning curve is commonly observed [11–15]. For example, reports of a learning curve for the modified Lapidus procedure and Minimally Invasive Chevron and Akin osteotomy (for hallux valgus) have been described in the literature [11, 12, 14, 16, 17]. Conversely, to the best of our knowledge, there does not seem to be any literature on the learning curve for the percutaneous ZO. Therefore, the purpose of the present study was to determine if there is a learning curve associated with the percutaneous ZO. If present, we hoped to evaluate the impact of this learning curve on patient outcomes and postoperative complications following percutaneous ZO.

## Methods

After institutional review board approval, a retrospective chart review was performed on patients who underwent percutaneous ZO between October 2017 and July 2021 at one institution. Surgery was performed by a fellowship-trained orthopaedic foot and ankle surgeon with notable MIS experience. However, these cases represent this surgeon’s first ZO cases, dating within a year of finishing fellowship. Percutaneous ZO was performed on patients presenting with IAT and Haglund deformity who had failed conservative management for greater than six months (Table 1). We consider the percutaneous ZO for all patients with IAT, and do not exclude patients based on any particular radiographic criteria. However, patients under 18 years of age and patients undergoing revision surgery with a percutaneous ZO (for failed open Haglund’s resection with Achilles reattachment) were excluded from this study.

**Table 1:** Patient characteristics

Age	51.28 ± 11.12 (range 28–81) years
**Sex**	61 females, 37 males
**Follow-up**	42.07 ± 12.99 (range 24–65) months

Surgical start and end times were recorded for all patients. All charts were reviewed for postoperative complications, revisions, and union rates at a minimum two-year follow-up. Functional Foot Index (FFI) scores were utilized to measure patients’ functional limitations and symptom presentation. Visual Analogue Scale (VAS) scores were utilized to measure patients’ pain presentation. Both measurements were taken at preoperative and each postoperative appointment. Scores at final follow-up were used for paired analysis.

### Surgical technique

**Fig. 1:**
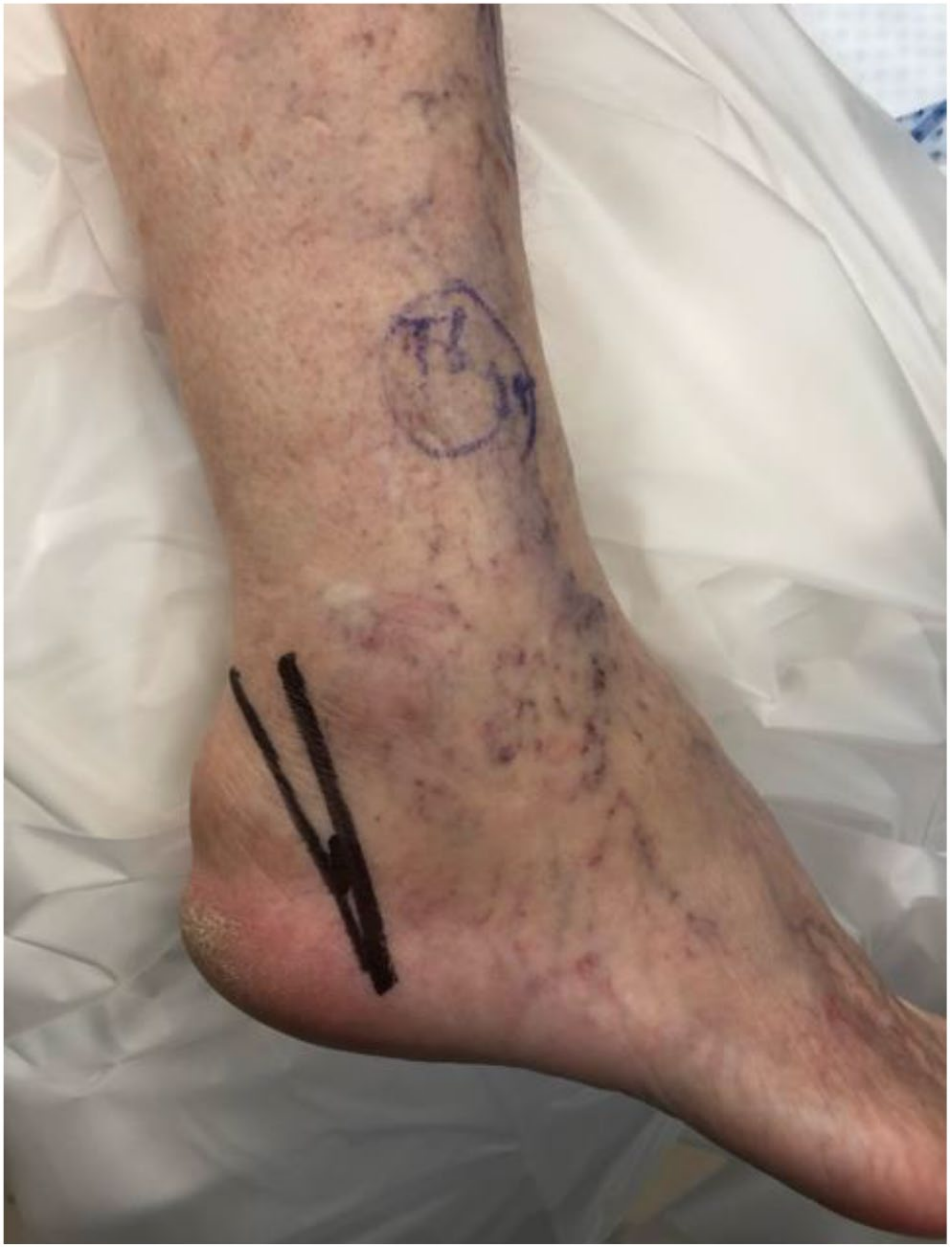
Preoperative, lateral clinical image of Haglund’s Deformity; marked prior to Zadek Osteotomy

All patients received the percutaneous ZO as previously described by Nordio et al. (Fig. 1) [2, 3]. Prior to surgery, all patients received a lower extremity popliteal and adductor canal or saphenous nerve block. The percutaneous ZO was performed under IV propofol sedation or general anesthesia. No tourniquet was used for the procedure. The patient was positioned in the lateral decubitus position with the operative leg off of the end of the bed resting on the mini C-arm. The nonoperative leg was flexed out of the field and taped to the bed.

**Fig. 2:**
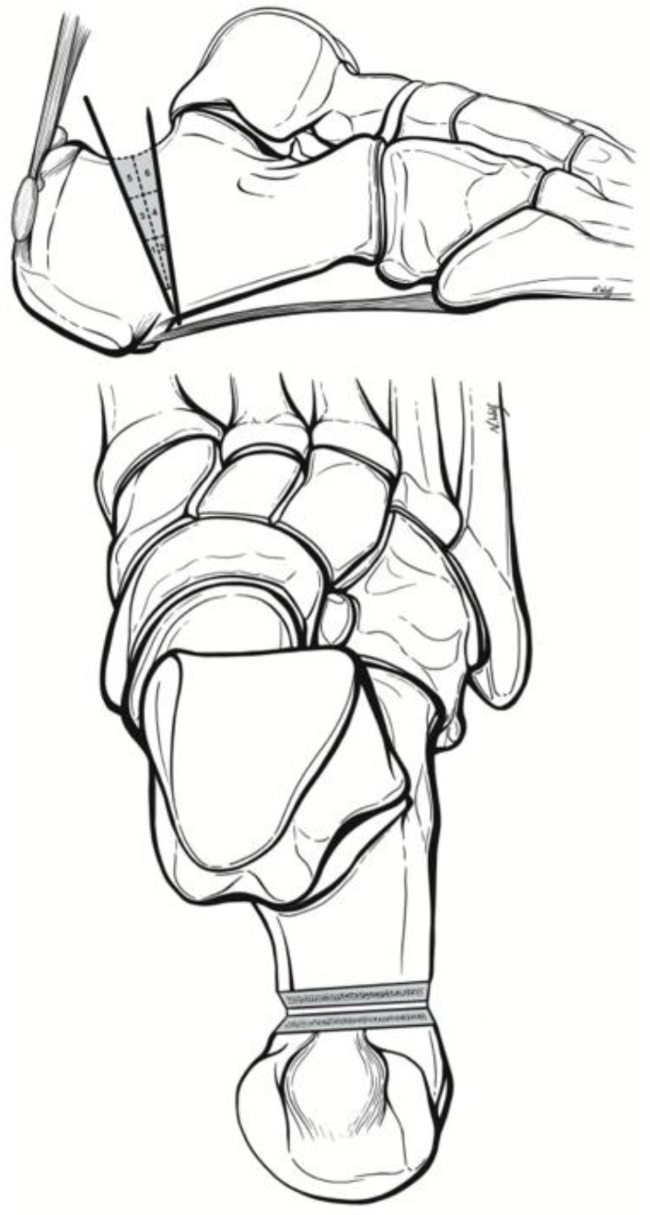
Surgical technique and quadrant cut guide as previously described by Kaplan et al [3].

A small 5 mm lateral calcaneal incision was made at the apex of the ZO, ~ 5–8 mm from the plantar cortex just anterior to the insertion of the plantar fascia at the calcaneal tuberosity. A blunt straight hemostat was used to spread down to bone prior to burr placement. A 3 mm x 30 mm Shannon burr (Novastep, Englewood Cliffs, New Jersey) was advanced into the lateral calcaneus at the apex of the osteotomy and its position was confirmed on lateral fluoroscopy view of the foot [3]. The osteotomy was then complete with Shannon burr. Copious cooled irrigation along with a pulsed burr technique was used throughout burring to reduce the heat generated at the osteotomy site, as described previously [18]. Great care was taken to maintaining a plantar cortical hinge of 5–8 mm of bone (Fig. 2).

The osteotomy was reduced with maximal ankle dorsiflexion to reduce the osteotomy. Two 7.0 mm headless compression screws were advanced across the osteotomy with the ankle in maximal dorsiflexion. Implant position was confirmed on lateral and Harris axial fluoroscopy views of the calcaneus. Incisions were irrigated with normal saline and closed with 3 − 0 nylon sutures. Patients are made immediately weightbearing as tolerated in a Controlled Ankle Motion boot. Patients were allowed to transition into normal shoes by six-weeks postoperation.

### Data analysis

**Fig. 3:**
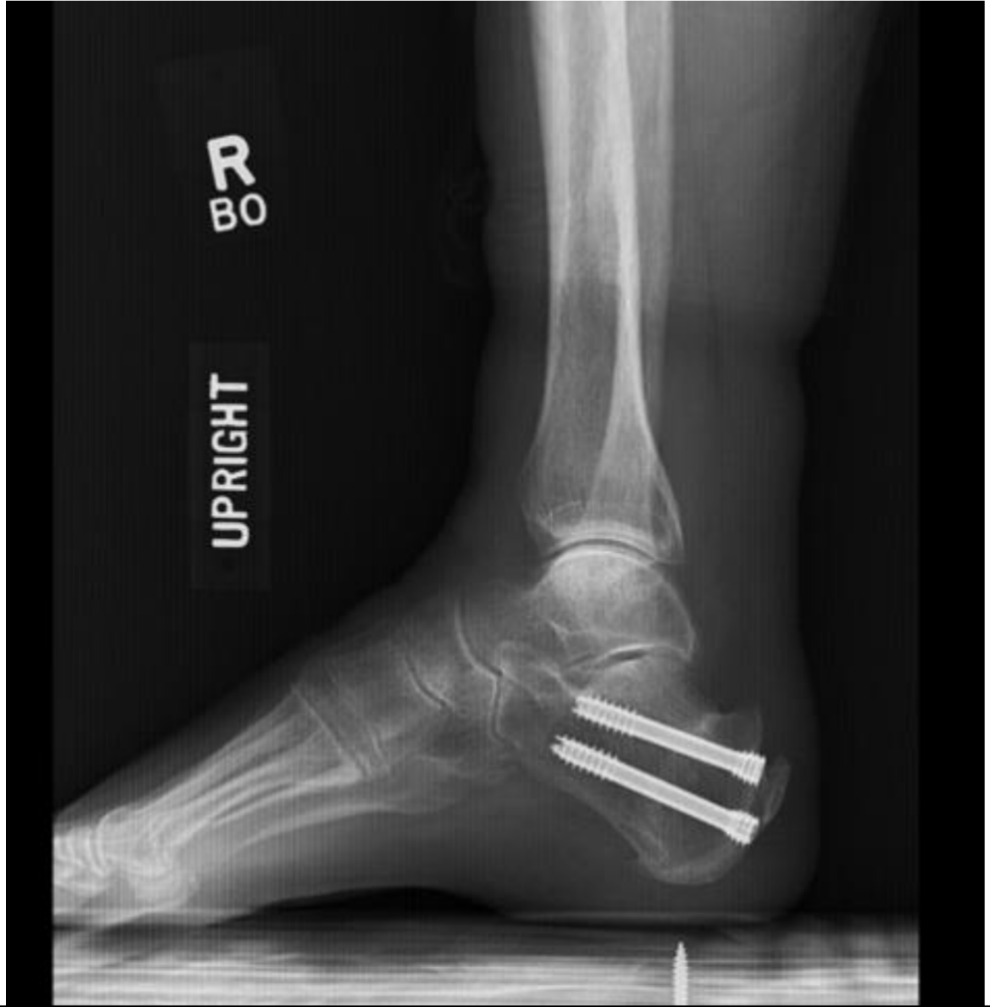
Postoperative lateral radiograph demonstrating two posterior screws and correction of Haglund’s deformity

FFI and VAS scores were collected at preoperative and postoperative follow-up appointments to measure patients’ functional limitations and pain presentation, respectively (Fig. 3). Change in FFI and VAS scores was calculated by subtracting final postoperative follow-up scores from preoperative (baseline) scores. A one-way ANOVA with Bonferroni post hoc analysis was performed to analyze differences in OR times, change in FFI scores, change in VAS scores, and rate of complication amongst intervals of cases as defined in Tables 2 and 3. IBM SPSS Statistics 28 (IBM, New York USA) software was used for all analysis.

**Table 2:** Results summary - cases grouped in chronological order

Group	1	2	3	4	5	6	7
Case Numbers	Cases 1–7	Cases 8–14	Cases 15–21	Cases 22–35	Cases 36–42	Cases 43–49	Cases 50 − 26
**OR Time (minutes)**	43.43 ± 2.76 (range 40–48)	36.43 ± 1.99 (range 33–39)	30.14 ± 3.13 (range 27–35)	26.57 ± 1.90 (range 24–29)	26.00 ± 1.83 (range 23–28)	23.00 ± 1.91 (range 21–27)	20.71 ± 1.50 (range 19–23)
**Complication**	0	2	0	0	0	0	1
**ΔFFI**	42.71 ± 3.04 (range 38–46)	46.57 ± 5.06 (range 41–54)	45.71 ± 3.25 (range 42–49)	42.29 ± 5.47 (range 35–50)	44.57 ± 4.04 (range 39–50)	44.71 ± 4.46 (range 40–51)	41.71 ± 17.88 (range 3–60)
**ΔVAS**	7.43 ± 1.27 (range 5–9)	7.14 ± 1.95 (range 5–10)	7.43 ± 1.40 (range 6–10)	6.86 ± 1.46 (range 5–9)	7.86 ± 1.34 (range 6–10)	7.43 ± 1.40 (range 6–10)	6.00 ± 2.45 (range 1–8)
**Follow-up (months)**	61.57 ± 2.15 (range 59–65)	58.00 ± 0.82 (range 57–59)	55.85 ± 0.69 (range 55–57)	53.57 ± 0.53 (range 53–54)	51.71 ± 0.76 (range 51–53)	49.00 ± 0.58 (range 48–50)	45.86 ± 0.69 (range 45–47)
**Group**	**8**	**9**	**10**	**11**	**12**	**13**	**14**
**Case Numbers**	**Cases 51–56**	**Cases 57–63**	**Cases 64–70**	**Cases 71–77**	**Cases 78–84**	**Cases 85–91**	**Cases 92–98**
**OR Time (minutes)**	20.43 ± 1.27 (range 19–22)	21.29 ± 1.11 (range 20–23)	23.14 ± 1.46 (range 21–25)	20.86 ± 3.13 (range 16–26)	22.14 ± 2.27 (range 20–27)	24.29 ± 3.73 (range 20–31)	24.14 ± 4.18 (range 19–31)
**Complication**	0	0	0	0	0	0	0
**ΔFFI**	45.71 ± 3.25 (range 42–49)	43.00 ± 4.62 (range 37–50)	42.86 ± 5.15 (range 37–50)	44.86 ± 4.67 (range 40–51)	46.00 ± 7.02 (range 37–60)	45.00 ± 4.93 (range 39–51)	45.00 ± 4.83 (range 38–52)
**ΔVAS**	7.29 ± 1.70 (range 5–10)	7.14 ± 1.46 (range 6–10)	7.86 ± 1.35 (range 6–10)	6.57 ± 0.98 (range 5–8)	7.71 ± 1.90 (range 6–10)	7.00 ± 1.00 (range 6–8)	8.00 ± 0.82 (range 7–9)
**Follow-up (months)**	42.29 ± 1.80 (range 40–45)	36.71 ± 1.80 (range 34–39)	30.86 ± 0.69 (range 30–32)	28.86 ± 1.07 (range 27–30)	25.23 ± 0.95 (range 24–27)	24.42 ± 0.53 (range 24–25)	25.00 ± 0.00 (range 25–25)

**Table 3:** ANOVA – analysis of operating room time between groups

Case Group	2	3	4	5	6	7	8	9	10	11	12	13	14
**1**	*p* < 0.001*	*p* < 0.001*	*p* < 0.001*	*p* < 0.001*	*p* < 0.001*	*p* < 0.001*	*p* < 0.001*	*p* < 0.001*	*p* < 0.001*	*p* < 0.001*	*p* < 0.001*	*p* < 0.001*	*p* < 0.001*
**2**		*p* = 0.001*	*p* < 0.001*	*p* < 0.001*	*p* < 0.001*	*p* < 0.001*	*p* < 0.001*	*p* < 0.001*	*p* < 0.001*	*p* < 0.001*	*p* < 0.001*	*p* < 0.001*	*p* < 0.001*
**3**			*p* = 0.758	*p* = 0.216	*p* < 0.001*	*p* < 0.001*	*p* < 0.001*	*p* < 0.001*	*p* < 0.001*	*p* < 0.001*	*p* < 0.001*	*p* = 0.003*	*p* = 0.002*
**4**				*p* = 1.00	*p* = 0.758	*P* = 0.003*	*p* = 0.001*	*p* = 0.012*	*p* = 1.00	*p* = 0.004*	*p* = 0.110	*p* = 1.00	*p* = 1.00
**5**					*p* = 1.00	*p* = 0.012*	*p* = 0.006*	*p* = 1.00	*p* = 1.00	*p* = 0.018*	*p* = 0.410	*p* = 1.00	*p* = 1.00
**6**						*p* = 1.00	*p* = 1.00	*p* = 1.00	*p* = 1.00	*p* = 1.00	*p* = 1.00	*p* = 1.00	*p* = 1.00
**7**							*p* = 1.00	*p* = 1.00	*p* = 1.00	*p* = 1.00	*p* = 1.00	*p* = 0.758	*p* = 1.00
**8**								*p* = 1.00	*p* = 1.00	*p* = 1.00	*p* = 1.00	*p* = 0.410	*p* = 0.560
**9**									*p* = 1.00	*p* = 1.00	*p* = 1.00	*p* = 1.00	*p* = 1.00
**10**										*p* = 1.00	*p* = 1.00	*p* = 1.00	*p* = 1.00
**11**											*p* = 1.00	*p* = 1.00	*p* = 1.00
**12**												*p* = 1.00	*p* = 1.00
**13**													*p* = 1.00

## Results

**Fig. 4:**
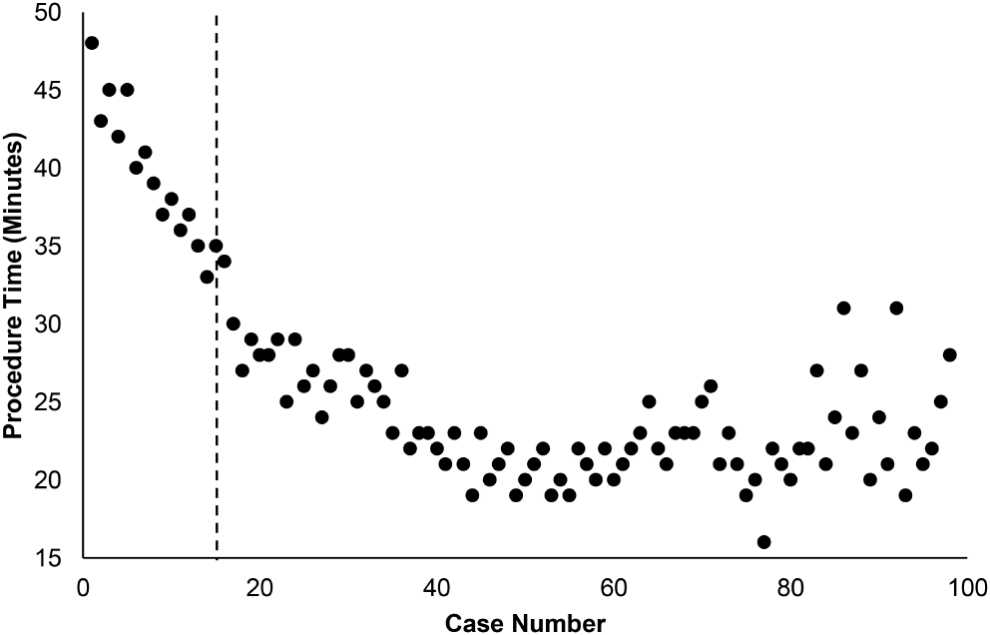
OR time vs. surgeon experience (case number). Cases decreased in duration significantly until case 14, as determined by ANOVA (*p* < 0.05, indicated by dotted line). At this point OR time plateaus, suggesting the learning curve has been overcome

A total of 98 consecutive percutaneous ZO procedures were retrospectively analyzed for this study. Participants included 61 females and 37 males. Mean age was 51.28 ± 11.12 (range 28–81) years; mean follow-up time was 42.07 ± 12.99 (range 24–65) months. A significant decrease in procedure time was detected between increasing case intervals (*p* < 0.05). This trend plateaued around case 14 (Fig. 4). An increase in experience, as a function of number of completed cases, was associated with a statistically significant decrease in surgery duration. For example, in the absence of percutaneous ZO experience, the mean duration of the procedure was 43.43 ± 2.76 (range 40–48) minutes. However, by the 14th case, the mean procedure duration decreased to 36.43 ± 1.99 (range 33–39) minutes.

One-way ANOVA with Bonferroni post hoc analysis revealed a statistically significant difference between operative times from cases 1–7 and cases 14–98 (*p* < 0.001). Further, ANOVA detected longer operative times amongst cases 1–14 in comparison to intervals of cases 15–98 (*p* < 0.001). Beyond our surgeons’ 14th case, the mean operative duration dropped to 27.57 ± 2.93 min (range 23–35; cases 15–35); the remainder of cases continued to plateau after this point. While the interval of cases 15–21 had shorter procedure times than cases 1–14 (*p* < 0.001), they were found to have longer operative times than cases 43–98 (*p* < 0.05). All statistical differences in procedure duration among intervals of cases are demonstrated in Table 3. Given the small sample size of each group, a Kruskal Wallis nonparametric test was also run. This supplementary analysis confirmed a statistically significant difference in operative time between the first two groups of cases (case 1–14) and the remaining cases (case 15–98), supporting the presence of the learning curve and location of its resolve.

**Fig. 5:**
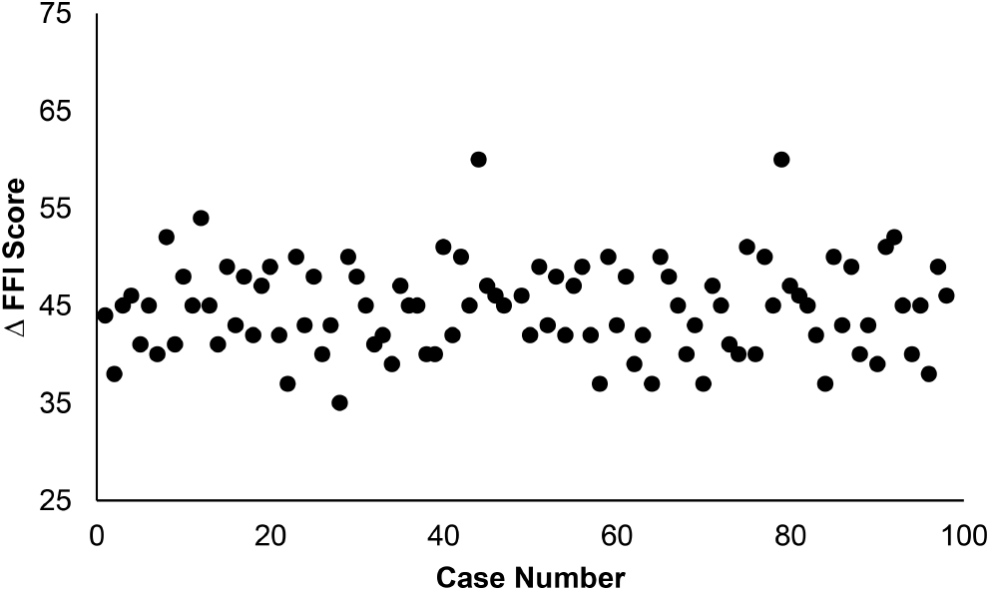
Postoperative change in Functional Foot Index (FFI) scores vs. increasing surgeon experience (case number). There was no statistical difference detected by ANOVA

**Fig. 6:**
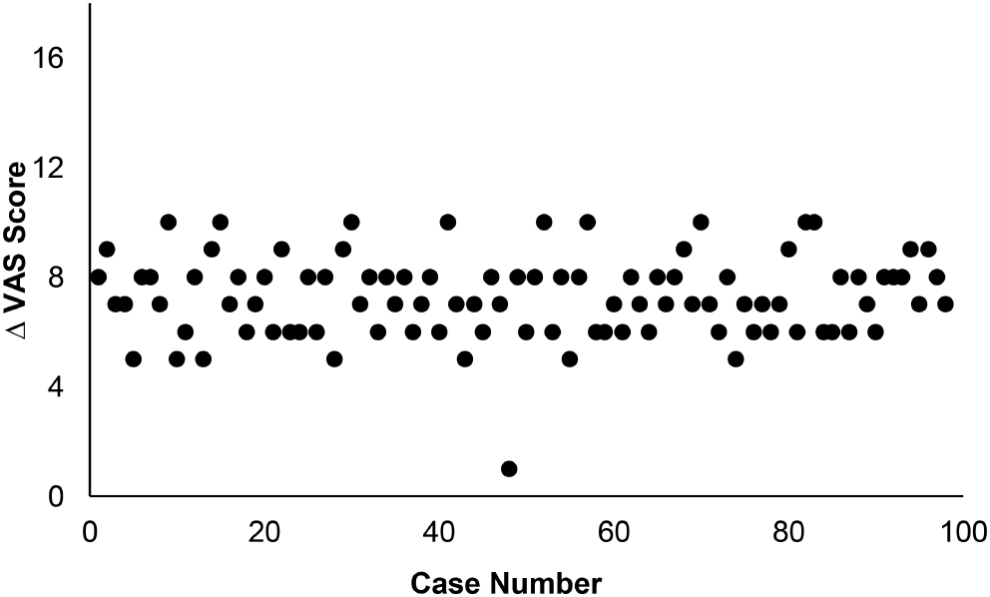
Postoperative change in Visual Analogue Scores (VAS) vs. increasing surgeon experience (case number). There was no statistical difference detected by ANOVA

In patients receiving percutaneous ZO, mean preoperative FFI scores was 55.31 ± 3.92 (range 48–67). Mean FFI score at final postoperative follow-up was 10.97 ± 5.20 (range 7–59). This was determined to be a statistically significant improvement by paired t-test and was observed within each case group (*p* < 0.001). Similarly, VAS scores improved significantly within each case group following percutaneous ZO. Preoperatively, mean VAS score was 7.67 ± 1.22 (range 6–10) amongst all patients. At final postoperative follow-up, mean VAS score improved to 0.41 ± 0.87 (range 0–7, *p* < 0.001). ANOVA demonstrated no statistical difference in the postoperative FFI (Fig. 5) nor VAS (Fig. 6) score improvement observed between intervals of cases. Across all 98 cases, we observed an overall complication rate of 3.06% (3/98). There was one case of nonunion (case 10) due to violation of the plantar hinge (1.02%). Separately, two patients experienced postoperative pain at the screw head which required hardware removal (2.04%; cases 13, 49). There was no statistical difference in complication rate detected between groups.

## Discussion

The present study first evaluated if a learning curve was associated with the percutaneous ZO. Our findings do indicate the presence of a learning curve with this MIS technique. Statistical analysis demonstrated procedure time to be inversely correlated with a surgeons’ experience with the technique, however this trend quickly plateaued. We do believe this statistically significant observation represents a period of a surgeon’s accommodation to comfortability with this procedure. As defined by significant changes in procedure duration, the learning curve associated with percutaneous ZO appears to be overcome around case 14.

While the learning curve may influence procedure duration, there were no other statistically significant observations related to a surgeon’s experience level. We detected no significant difference in complications observed before and after this learning curve resolved. Further, there was no difference in complication rate detected between individual groups of cases. Additionally, patient reported outcome scores were similar before and after the resolution of the learning curve. While each group demonstrated a significant improvement in patient reported function and pain following percutaneous ZO intervention (FFI and VAS scores, respectively), we detected no differences in the postoperative change in FFI or VAS scores observed between groups of cases.

Minimally invasive approaches are gaining significant traction in foot and ankle surgery, with some of these procedures requiring a surgeon to overcome a learning curve [19]. MIS procedures in foot and ankle orthopedics are commonly associated with a significant learning curve. More specifically, learning curves for the modified, percutaneous subcapital Bösch osteotomy [15], the modified Lapidus procedure [13], the minimally invasive Chevron-Akin (MICA) [11, 14, 17, 20, 21], , the single-screw percutaneous MICA [12], and the percutaneous double first metatarsal osteotomy (PEDO) [22], and have been presented in the literature recently.

In a three-year, retrospective study with cases of one surgeon, Ghioldi et al. described a significant learning curve associated with the modified, percutaneous subcapital Bösch Osteotomy. This learning curve was overcome by the 30th case [15]. In a separate retrospective study, Jackson et al. described a learning curve associated with the modified Lapidus procedure. They reported resolution of this learning curve by case 23 [13]. Jowett and Bedi first described a learning curve associated with MICA, which was comparable to that of the procedure’s open counterpart [11]. This observation was confirmed by a retrospective study by Palmanovich et al. [12]. , prospective study by Toepfer and Strässle [14], and most recently by Lewis et al. [16] Further, the learning curves described by Palmanovich et al., Toepfer and Strässle, and Lewis et al. did not demonstrate impact on complication rates. These learning curves were overcome by case 27, 40, and 38 respectively [12, 14, 16]. 

In the present study, we observed a learning curve associated with the percutaneous ZO, which resolved around case 14. It should again be noted that the operating surgeon in the current study is fellowship trained in foot and ankle surgery and had a foundation of MIS technique prior to the cases presented in this study. Proper MIS training and technique should be utilized when learning any new MIS procedure. Nevertheless, our data suggests a slightly shorter learning curve for the ZO technique in comparison to other percutaneous procedures [11, 12, 14, 16]. While a formal comparison of these learning curves may not be clinically relevant, we present this loosely as a reference to attest to the learnability of the ZO technique. Additionally, while there were very few complications observed in this case series (3.06%), there was also no statistical difference detected in complication rate between any two groups of cases. Therefore, according to our current data, this learning curve has no detectable impact on complication rate. Similarly, significant improvements in FFI and VAS scores were observed at final follow-up within each case interval (*p* < 0.001). There was no statistical difference detected between the magnitude of FFI and VAS improvement in relation to a surgeon’s experience (Figs. 2 and 3).

Limitations of this study include its retrospective nature, which carries inherent flaws. As formal preoperative and postoperative range of motion, strength, or biomechanical measurements are not a part of routine standard of care, they are not represented in the current study. Additionally, the surgeon studied in the current analysis has extensive MIS experience, potentially limiting the generalizability of our findings. However, these cases represent the surgeons first 98 ZO cases, within a year of finishing fellowship. Further, by analyzing complications, patient reported outcomes, and operative time in relation to increasing case number in a large volume of cases, we were still able to characterize a period of accommodation to comfortability with the percutaneous ZO procedure. Simultaneously, we were able to provide more evidence that this technique is a safe and effective treatment of IAT, even in a surgeon’s first few cases. A larger, multicenter prospective study assessing multiple surgeons with various levels of MIS experience is warranted for further evaluation and broadened generalizability of our findings.

## Conclusion

There was a learning curve associated with the percutaneous ZO. While statistically significant, this learning curve was only observed in regard to procedure length and was overcome around case 14. Further, a surgeon’s inexperience with this technique does not appear to negatively influence patient outcomes, nonunion rates, or rate of revision. Additionally, there was no increase in complications observed with this learning curve. The percutaneous ZO appears to be a safe and effective treatment of IAT and HD, regardless of a surgeon’s level of experience with the technique.

### Electronic supplementary material

Below is the link to the electronic supplementary material.

Supplementary Material 1

## Data Availability

The data that support the findings of this study are available on request from the corresponding author.
